# IL-15 promotes self-renewal of progenitor exhausted CD8 T cells during persistent antigenic stimulation

**DOI:** 10.3389/fimmu.2023.1117092

**Published:** 2023-06-20

**Authors:** Junghwa Lee, Kyungmin Lee, Hyeonjin Bae, Kunhee Lee, Solhwi Lee, Junhui Ma, Kyungjo Jo, Ijun Kim, ByulA Jee, Minyong Kang, Se Jin Im

**Affiliations:** ^1^ Department of Precision Medicine, Sungkyunkwan University School of Medicine, Suwon, Republic of Korea; ^2^ Department of Immunology, Graduate School of Basic Medical Science, Sungkyunkwan University School of Medicine, Suwon, Republic of Korea; ^3^ Department of Urology, Samsung Medical Center, Sungkyunkwan University School of Medicine, Seoul, Republic of Korea; ^4^ Department of Health Sciences and Technology, SAIHST, Sungkyunkwan University, Seoul, Republic of Korea; ^5^ Samsung Genome Institute, Samsung Medical Center, Seoul, Republic of Korea

**Keywords:** progenitor exhausted CD8 T cells, maintenance, IL-15, chronic viral infection, cancer

## Abstract

In chronic infections and cancer, exhausted CD8 T cells exhibit heterogeneous subpopulations. TCF1+PD-1+ progenitor exhausted CD8 T cells (Tpex) can self-renew and give rise to Tim-3+PD-1+ terminally differentiated CD8 T cells that retain their effector functions. Tpex cells are thus essential to maintaining a pool of antigen-specific CD8 T cells during persistent antigenic stimulation, and only they respond to PD-1-targeted therapy. Despite their potential as a crucial therapeutic target for immune interventions, the mechanisms controlling the maintenance of virus-specific Tpex cells remain to be determined. We observed approximately 10-fold fewer Tpex cells in the spleens of mice chronically infected with lymphocytic choriomeningitis virus (LCMV) one-year post-infection (p.i.) than at three months p.i. Similar to memory CD8 T cells, Tpex cells have been found to undergo self-renewal in the lymphoid organs, prominently the bone marrow, during chronic LCMV infection. Furthermore, *ex vivo* treatment with IL-15 preferentially induced the proliferation of Tpex cells rather than the terminally differentiated subsets. Interestingly, single-cell RNA sequencing analysis of LCMV-specific exhausted CD8 T cells after *ex vivo* IL-15 treatment compared with those before treatment revealed increased expression of ribosome-related genes and decreased expression of genes associated with the TCR signaling pathway and apoptosis in both Tpex and Ttex subsets. The exogenous administration of IL-15 to chronically LCMV-infected mice also significantly increased self-renewal of Tpex cells in the spleen and bone marrow. In addition, we assessed the responsiveness of CD8 tumor-infiltrating lymphocytes (TILs) from renal cell carcinoma patients to IL-15. Similar to the data we obtained from chronic viral infection in mice, the expansion of the Tpex subset of PD-1+ CD8 TILs upon *ex vivo* IL-15 treatment was significantly higher than that of the terminally differentiated subset. These results show that IL-15 could promote self-renewal of Tpex cells, which has important therapeutic implications.

## Introduction

T cell exhaustion arises during chronic infections and cancer and has now become a target of immunotherapy. In the environments with persisting antigens, antigen-specific CD8 T cells become dysfunctional, gradually losing their effector functions and proliferative capacity and acquiring multiple inhibitory receptors, such as programmed cell death-1 (PD-1) (1, 2). Recent studies, including our first discovery, demonstrated that exhausted CD8 T cells are composed of heterogeneous subpopulations (3–10). Tpex cells, which could be identified by their exclusive expression of T cell factor 1 (TCF1), CXC-chemokine receptor 5 (CXCR5), signaling lymphocytic activation molecule (SLAM) family member 6 (Slamf6), or the ectonucleotidase CD73, are capable of self-renewal and give rise to T cell immunoglobulin and mucin domain–containing protein 3 (Tim-3)+PD-1+ terminally differentiated CD8 T cells that retain their effector functions but persist for only a short period. Following antigen stimulation, Tpex cells can proliferate and differentiate into transitory exhausted CD8 T cells (Ttrex, Tim-3+CD101-), which exhibit the strongest cytolytic activity among the subsets, and subsequently to terminally exhausted CD8 T cells (Ttex, Tim-3+CD101+), which become dysfunctional (3–11). Using *Tcf7*-deficient CD8 T cells, it has been verified that Tpex cells are essential to maintaining antigen-specific CD8 T cell responses during chronic antigen exposure (3, 5). Furthermore, it is worth noting that only this subset of exhausted CD8 T cells proliferates in response to αPD-1 therapy in animal models (3–5, 9, 10, 12). Preclinical studies have demonstrated that Tpex cells can control chronic viral infection and tumor growth better than Ttex cells. Transfer of CD44^hi^ CXCR5+ CD8 T cells into mice chronically infected with LCMV resulted in significantly improved viral control compared with the use of CD44^hi^ CXCR5- CD8 T cells (4). B16-ovalbumin (OVA) bearing mice that received the progenitor subset of CD8 TILs showed substantially reduced tumor growth compared with mice treated with the terminally exhausted subset (9). Furthermore, the presence of tumor-infiltrating TCF1+ CD8 T cells in the tumors of melanoma patients correlated with the responsiveness to αPD-1 therapy (13), and a higher frequency of Tpex cells among the PD-1+ CD8 TILs was associated with prolonged progression-free survival and overall survival in patients with melanoma who received immune checkpoint blockade therapy (9). Thus, Tpex cells are the major target of immunotherapeutic interventions against chronic viral infections and cancer, but the mechanisms underlying the maintenance of Tpex cells remain largely unknown.

During chronic viral infection, exhausted CD8 T cells are not efficiently maintained in the absence of the antigen, which is different from the behavior of conventional memory CD8 T cells developed after an acute viral infection (14, 15). Moreover, exhausted CD8 T cells have an impaired ability to proliferate in response to *ex vivo* interleukin-7 (IL-7) or IL-15 treatment and appeared not to need those cytokine-mediated signals to be sustained *in vivo* (14). Therefore, it has been perceived that virus-specific exhausted CD8 T cells require cognate antigen stimulation to persist during chronic infection (15). However, given their heterogeneity, the overall maintenance of exhausted CD8 T cells could reflect the relatively large population of terminally differentiated cells without adequately reflecting the characteristics of the small proportion of Tpex cells. Considering the differential functional properties of Tpex cells and other exhausted CD8 T cell subpopulations, the mechanisms underlying the maintenance of Tpex cells need to be reevaluated. Utzschneider et al. showed that a subset of exhausted CD8 T cells could persist for 4 weeks after antigen removal and re-expand upon antigen encounter (16). Additionally, although exhausted CD8 T cells isolated from chronically LCMV-infected mice in a previous report declined dramatically in an antigen-free environment, some cells remained (14). Likewise, most virus-specific, exhausted CD8 T cells did not respond to *ex vivo* treatment with IL-7 or IL-15, but a small fraction of cells was observed to divide in response to those cytokines. Those results imply that Tpex cells might be maintained by homeostatic cytokines rather than antigenic stimulation, which is one underlying mechanism for sustaining conventional memory CD8 T cells generated by acute viral infections and vaccination.

The IL-15 receptor complex consists of a distinctive α chain (CD215), IL-2/IL-15 receptor β chain (CD122), and common γ chain (γ_c_, CD132) (17, 18). Because the main mechanism of IL-15 is trans-presentation, whereby the IL-15 and IL-15Rα expressed by the same cells bind and transduce signals to the β/γ chains of IL-15 receptor-expressing neighbor cells, the availability and responses of IL-15 depend on the cellular source of it and the anatomical site. In the homeostatic state or following viral infection, CD8α+ dendritic cells have been reported as major cell types producing IL-15 (19). Of interest, CD8α+ dendritic cells specifically express a chemokine receptor for XCL1, XCR1 (20), and the Tpex subset exhibited a high level of *Xcl1* mRNA expression (3). Furthermore, it was recently presented that Tpex cells preferentially reside in an antigen-presenting cells (APC)-enriched area of the spleen in chronically LCMV-infected mice where CD8α+ conventional type 1 dendritic cells provide a specific niche to preserve Tpex cells and protect them from activation and further differentiation (21). In a murine tumor microenvironment, it has been shown that myeloid cells including monocytes, granulocytes, and macrophages predominantly express IL-15 (22). Analogous to those data, it has also been reported that the presence of Tpex cells in the human tumor microenvironment requires a specialized niche enriched with APCs (8). These observations suggest that, during the interaction with APCs, IL-15 could be one of the mechanisms that maintain Tpex cells under chronic antigenic stimulation.

Following antigen clearance, memory CD8 T cells persist in an antigen-independent manner *via* homeostatic proliferation driven by IL-7 and IL-15 (23, 24). IL-15-mediated homeostatic proliferation, which occurs predominantly in the bone marrow, is essential for the maintenance of memory CD8 T cells (25–27). IL-15 also plays a crucial role in the development, function, and homeostasis of multiple immune cells, including natural killer (NK) cells (17, 28). Because it preferentially expands CD8 T cells and NK cells, diverse forms of IL-15 have been developed as immunotherapeutic agents to stimulate anti-tumor immune responses, and IL-15 has been explored in preclinical and clinical studies as a monotherapy and a combination treatment with other therapeutic interventions (17, 18, 28–30).

Given the similarity between the molecular signature and functional properties of Tpex cells and those of memory CD8 T cells (3, 5, 6), we here investigated the kinetics of Tpex cells and their IL-15-driven homeostatic proliferation during persistent antigenic stimulation. During chronic LCMV infection, virus-specific Tpex cells underwent self-renewal in the lymphoid organs, particularly the bone marrow, although their population was significantly reduced one-year p.i. Furthermore, we found that *ex vivo* and *in vivo* IL-15 treatment preferentially enhanced the self-renewal of Tpex cells during chronic LCMV infection. In addition, *ex vivo* IL-15 treatment of LCMV-specific exhausted CD8 T cells resulted in increased expression of genes encoding ribosomal proteins and decreased expression of genes involved in the T cell receptor (TCR) signaling pathway and apoptosis similarly in both Tpex and Ttex subsets. Consistently, the Tpex subset of PD-1+ CD8 TILs from human renal cell carcinoma (RCC) tumors also had a superior ability to proliferate following *ex vivo* IL-15 treatment, compared with the Ttex subset. Therefore, our results indicate that IL-15 could promote the self-renewal of Tpex cells during persistent antigenic stimulation, identifying a potential mechanism that supports the maintenance of Tpex cells.

## Materials and methods

### Mice, infections, and *in vivo* IL-15 administration

Six- to eight-week-old female C57BL/6 mice were purchased from the Jackson Laboratory (Bar Harbor, ME) and Orient Bio (Seongnam, Republic of Korea). For chronic LCMV infection, mice were intravenously infected with 2 x 10^6^ plaque-forming units of LCMV clone 13. Transient CD4 T cell depletion was performed at the onset of infection by injecting 200 μg of anti-CD4 depleting antibodies (GK1.5, BioXCell, Lebanon, NH) intraperitoneally (i.p.) two days prior to and on the day of infection. For all experiments, the chronically LCMV-infected mice were used 45 days p.i. For acute LCMV infection, mice were i.p. infected with 2 x 10^5^ plaque-forming units of LCMV Armstrong. The chronically LCMV-infected mice were i.p. treated with 5 μg of recombinant murine IL-15 (Peprotech, Rocky Hill, NJ) or phosphate-buffered saline (PBS) daily for three consecutive days as previously reported (14). All experiments were conducted in accordance with the Institutional Animal Care and Use Committee guidelines of the Sungkyunkwan University School of Medicine.

### Human RCC study subjects

Human TILs were prospectively obtained from the primary tumor tissue of patients with RCC who underwent radical nephrectomy at Samsung Medical Center in Seoul, and peripheral blood mononuclear cells (PBMCs) were collected on the day of surgery. The TILs and PBMCs were isolated as described previously (31). All methods regarding human resources were conducted in accordance with the Declaration of Helsinki, and the protocol was approved by the Institutional Review Board at Samsung Medical Center (approval number: 2020-03-063). All patients participating in this study provided written informed consent, and any personally identifiable information was completely removed using an anonymized processing protocol.

### Flow cytometry

Lymphocytes were isolated from multiple tissues and blood as described previously (26, 32). Major histocompatibility complex class I tetramers were prepared and used as previously described (33). All antibodies were purchased from BD Biosciences (San Jose, CA), Biolegend (San Diego, CA), Invitrogen (Carlsbad, CA), or Cell Signaling Technology (Danvers, MA). Anti-mouse antibodies used for flow cytometry included CD8 (clone 53-6.7, catalog 557654, BD Biosciences), CD44 (clone IM7, catalog 553133, BD Biosciences), CD101 (clone Moushi101, catalog 25-1011-82, Invitrogen), CD122 (clone TM-β1, catalog 564762, BD Biosciences), CD132 (clone TUGm2, catalog 132305, Biolegend), CXCR5 (clone L138D7, catalog 145504, Biolegend), PD-1 (clone 29F.1A12, catalog 135220, Biolegend), and Tim-3 (clone RMT3-23, catalog 119727, Biolegend). Anti-human antibodies used for flow cytometry included CD3 (clone OKT3, catalog 317322, Biolegend), CD8 (clone HIT8α, catalog 566855, BD Biosciences), CD28 (clone CD28.2, catalog 562296, BD Biosciences), CD39 (clone A1, catalog 328218, Biolegend), CD122 (clone Mik- β3, catalog 562887, BD Biosciences), CD132 (clone TUGh4, catalog 743443, BD Biosciences), PD-1 (clone EH12.2H7, catalog 329918, Biolegend), Tim-3 (clone F38-2E2, catalog 345024, Biolegend), and Ki-67 (clone B56, catalog 556026, BD Biosciences). TCF1 staining was performed using unconjugated TCF1 antibody (clone C63D9, catalog 2203S, Cell Signaling Technology) and fluorophore-conjugated goat anti-rabbit IgG secondary antibodies. Transcription factors were stained using a FoxP3 transcription factor staining buffer set (Invitrogen). 5-Bromo-2’-deoxyuridine (BrdU) incorporation was assessed using a BrdU flow kit (BD Biosciences) according to the manufacturer’s guidelines. Dead cells were excluded using Live/Dead fixable dead cell stain kits (Invitrogen). All data were acquired on a Cytoflex flow cytometer (Beckman Coulter, Brea, CA) and analyzed using FlowJo software (Tree Star, Ashland, OR).

### *In vivo* BrdU incorporation

For long-term BrdU treatment, chronically LCMV-infected mice were continuously provided with BrdU (BD Biosciences) at 0.8 mg/ml in their drinking water for 28 days. For the *in vivo* IL-15 administration experiments, chronically LCMV-infected mice i.p. received 2 mg of BrdU daily for three consecutive days. The day after the last treatment, the mice were sacrificed for analysis.

### *Ex vivo* proliferation assay

Lymphocytes were isolated from the spleens and bone marrow of chronically LCMV-infected mice and Armstrong immune mice. CD8 T cells were purified from splenocytes using a mouse CD8α+ T cell isolation kit (Miltenyi Biotech, San Diego, CA), labeled with CellTrace Violet (CTV) (Invitrogen), and mixed 1:1 with unlabeled naive splenocytes that were used as feeder cells. The total lymphocytes isolated from the bone marrow were CTV-labeled and mixed with unlabeled naive splenocytes (10% bone marrow cells). In the experiments with isolated T cell subsets, Tpex (live CD8+PD-1+CD73+Tim-3-) and Ttex (live CD8+PD-1+CD73-Tim-3+) cells were sorted from the CTV-labeled splenocytes of chronically LCMV-infected mice using BD FACSAria™ III Flow Cytometer (BD Biosciences) of the BIORP. Sorted cells were mixed with 5 x 10^5^ naïve splenocytes that were used as feeder cells and cultured in the presence and absence of recombinant murine IL-15 (Peprotech) at 100 ng/ml. After 3 days of culture, the cells were harvested and analyzed by flow cytometry. Cryopreserved TILs isolated from human RCC tumors and matched PBMCs were thawed and cultured at a 1:1 ratio in the presence of recombinant human IL-15 (Peprotech) at 10 ng/ml for 3 days. The cells were then collected and analyzed by flow cytometry. Autologous PBMCs were irradiated (25 Gy) and labeled with CTV to distinguish them before they were mixed with TILs.

### Single-cell RNA sequencing and analysis

Purified CD8 T cells from the spleens of chronically LCMV-infected mice were cultured with recombinant murine IL-15 (Peprotech) for 3 days as described above. From pre- and post- IL-15-cultured cells, live CD8+PD-1+(GP33+GP276)+ cells were sorted using BD FACSAria™ III Flow Cytometer (BD Biosciences) of the BIORP. Naïve CD8 T cells (live CD8+PD-1-CD44^lo^CD62L+) were sorted from the spleens of naïve mice and included as a control for analysis. Libraries were prepared using the Chromium controller according to the 10x chromium Next GEM Single Cell 3’ v3.1 protocol (CG000315). Briefly, the cell suspensions were diluted in nuclease-free water to achieve a targeted cell count of 5,000. The cell suspension was mixed with master mix and loaded with Single Cell 3’ v3.1 Gel Beads and Partitioning Oil into a chromium Next GEM chip G. RNA transcripts from single cells were uniquely barcoded and reverse-transcribed within droplets. cDNA molecules were pooled and went through an end repair process, the addition of a single ‘A’ base, and ligation of the adapters. The products are then purified and enriched with polymerase chain reaction (PCR) to create the final cDNA library. The purified libraries were quantified using quantitative PCR according to the quantitative PCR Quantification Protocol Guide (KAPA) and qualified using the Agilent Technologies 4200 TapeStation (Agilent technologies, Santa Clara, CA). The libraries were sequenced using HiSeq platform (Illumina, San Diego, CA) according to the read length in the user guide.

The processed single-cell RNA sequencing (scRNA-seq) data were aligned, filtered, and quantified using the Cell Ranger (v7.0.1, 10x Genomics, Pleasanton, CA). Further quality control and transcriptome analysis of the output data were performed using the Seurat package (v4.1.1). Doublets and low-quality cells were excluded from subsequent analysis (< 500 or > 6000 genes). The object was processed using NormalizedData, FindVariableFeatures, and ScaleData, followed by RunPCA and RunUMAP. Louvain clustering was applied to identify each cell cluster. To identify differentially expressed marker genes for the clusters, the function FindAllMarkers from the Seurat package was used with default parameters. To identify differentially expressed genes (DEGs) between two groups, the function FindMarkers from the Seurat package was conducted. To perform gene set enrichment analysis (GSEA), the Hallmark gene sets from the Molecular Signatures Database were used (https://www.gsea-msigdb.org/). GSEA was computed using the “fgsea” package (v1.22.0).

### Statistical analysis

All statistical analyses were performed using Prism 8 software (GraphPad, San Diego, CA). Statistical significance was determined by paired or unpaired two-tailed Student’s t-tests and one-way ANOVA as indicated. P values less than 0.05 or 0.01 were considered statistically significant.

### Data availability

ScRNA-seq data are available from GEO under accession number GSE233388.

## Results

### Tpex cells undergo self-renewal in the lymphoid organs, primarily the bone marrow, during chronic LCMV infection

**Figure 1:**
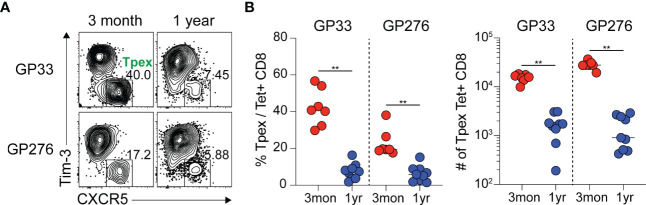
Virus-specific Tpex cells decrease over time during the course of chronic LCMV infection. **(A, B)** Representative flow plots **(A)** and summary graphs **(B)** showing the frequency and total number of Tpex (CXCR5+Tim-3-) cells among GP33- or GP276-specific CD8 T cells in the spleen at the indicated time points of chronic LCMV infection. Data were combined from two independent experiments with n=4–5 mice per group per experiment. The graphs show the mean and standard error of the mean (SEM). **P < 0.01 (Student’s *t*-test).

During chronic viral infection, virus-specific CD8 T cells are known to persist for extended periods, although they are functionally impaired (34). Because Tpex cells act as resource cells through their ability to reproduce themselves and generate terminally differentiated progeny, we wanted to investigate how virus-specific Tpex cells are sustained long-term during chronic infection. For this study, we used a chronic LCMV infection model in which CD4 T cells were transiently depleted prior to clone 13 infection to induce lifelong viremia, and we followed the GP33- and GP276-specific CD8 T cell responses for one-year p.i. in the spleen, where a high frequency of Tpex cells had already been observed during chronic LCMV infection. At one-year p.i., the Tpex subset of GP33- or GP276-specific CD8 T cells was approximately 10-fold smaller than it had been three months p.i. (**Figure 1**), indicating that virus-specific Tpex cells decreased over time in lymphoid organs such as the spleen during the course of a chronic infection, although they were sustained at a certain level for long periods.

**Figure 2:**
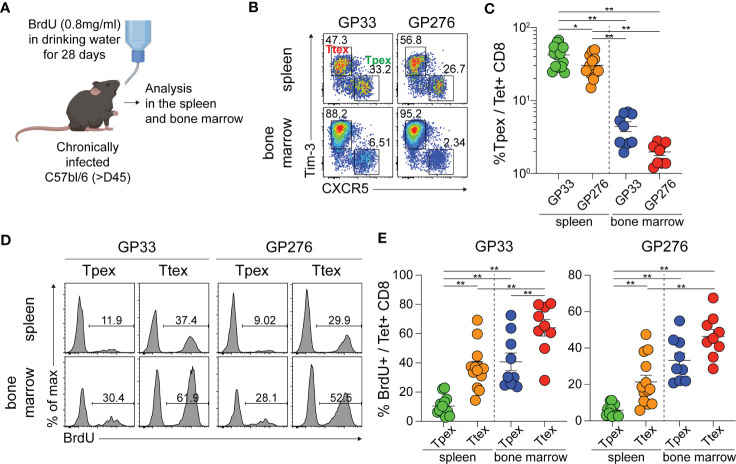
Tpex cells undergo self-renewal in the lymphoid organs, prominently in the bone marrow, during chronic LCMV infection. **(A)** Experimental setup: Chronically LCMV-infected mice (>day 45 p.i.) were given BrdU in their drinking water (0.8 mg/ml) for 28 days. **(B, C)** Representative flow plots **(B)** and a summary graph **(C)** showing the frequency of the Tpex (CXCR5+Tim-3-) and Ttex (CXCR5-Tim-3+) subsets of GP33- or GP276-specific CD8 T cells in the spleen and bone marrow after 28 days of BrdU incorporation. **(D, E)** Representative flow plots **(D)** and summary graphs **(E)** showing the frequency of BrdU+ cells in the Tpex and Ttex subsets from the spleen and bone marrow. Data were combined from two or three independent experiments with n=4–5 mice per group per experiment. The graphs show the mean and SEM. *P < 0.05; **P < 0.01 (one-way ANOVA).

Next, we examined where Tpex cells undergo proliferation to determine whether there is a preferential site for their self-renewal during persistent antigenic stimulation. Chronically LCMV-infected mice were continuously provided with BrdU in their drinking water to mark the proliferating cells (**Figure 2A**). We initially observed minimal self-renewal of Tpex cells after 7 days of BrdU incorporation (data not shown) and therefore extended the BrdU supply to 28 days. Because the Tpex subset was primarily localized in the lymphoid tissues such as the spleen and bone marrow (3, 35), we focused on these tissues. Of note, the frequency of Tpex cells among virus-specific CD8 T cells was higher in the spleen than in the bone marrow (**Figures 2B, C**). Among the GP33-specific CD8 T cells in the spleen, around 10% of Tpex cells had proliferated during the 28 days, and the proportion of proliferating cells in the Ttex subset was much higher (~40%) than that in the Tpex subset (**Figures 2D, E**). Considering the limited proliferative potential of Ttex cells (3), their high BrdU incorporation rates could result from the conversion of the Tpex subset into terminally differentiated cells upon antigenic stimulation, not reflecting self-renewal. Interestingly, we found that a significantly higher frequency (~40%) of GP33-specific Tpex cells from bone marrow had incorporated BrdU, compared with those in the spleen, showing that Tpex cells undergo self-renewal predominantly in the bone marrow. Also, higher percentages of BrdU+ cells were observed among the Ttex subset in the bone marrow, compared with those in the spleen. The same results were seen with GP276-specific CD8 T cells. Therefore, it appears that although the Tpex subset undergoes self-renewal in both the spleen and bone marrow, bone marrow is the major site where Tpex cells reproduce themselves and replenish exhausted CD8 T cells during chronic viral infection, which is similar to memory CD8 T cells, whose homeostatic self-renewal occurs mainly in the bone marrow (27).

### *Ex vivo* treatment of IL-15 preferentially enhances the proliferation of virus-specific Tpex cells

**Figure 3:**
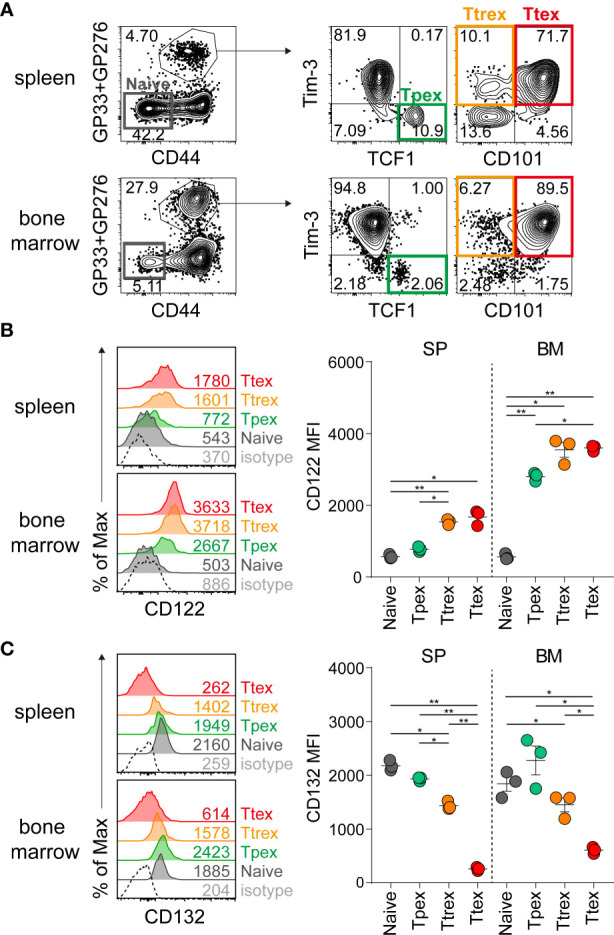
Expression of IL-15 receptor subunits on virus-specific CD8 T cells during chronic LCMV infection. **(A)** Identification of Tpex (TCF1+Tim-3-), Ttrex (Tim-3+CD101-) and Ttex (Tim-3+CD101+) subsets among GP33+GP276-specific CD8 T cells and a naïve (CD44^lo^PD-1-) population in the spleen and bone marrow from the same host during chronic LCMV infection (>day 45 p.i.). **(B, C)** Expression of CD122 **(B)** and CD132 **(C)** on each subpopulation of CD8 T cells given in **(A)**. Data are representative of two independent experiments with n=3 mice per group per experiment. The graphs show the mean and SEM. *P < 0.05; **P < 0.01 (one-way ANOVA). Statistical significance was determined for the comparison between four subsets in each tissue. MFI, mean fluorescent intensity; SP, spleen; BM, bone marrow.

We next investigated whether IL-15 treatment would lead to the homeostatic proliferation of Tpex cells, similar to memory CD8 T cells (25, 26). Before assessing the responsiveness of virus-specific exhausted CD8 T cell subsets to IL-15, we first analyzed their expression of CD122 and CD132, which are the beta and gamma chains of the IL-15 receptor and responsible for IL-15-mediated signaling, during chronic LCMV infection. The virus-specific Tpex subset was defined with the expression of a transcription factor, TCF1, and Tim-3+ terminally differentiated T cells were further divided into CD101- Ttrex and CD101+ Ttex cells (**Figure 3A**). As previously reported in mice (36), naïve CD8 T cells express minimal CD122 (**Figure 3B**). During chronic viral infection, CD122 was upregulated on LCMV-specific CD8 T cells, compared with naïve CD8 T cells, and there was a trend toward higher levels of CD122 expression on the Ttrex and Ttex subsets than on Tpex cells. The same results were obtained in exhausted CD8 T cells from the spleen and bone marrow, but it is noteworthy that the expression of CD122 was higher in the bone marrow than in the spleen. In agreement with previous studies (37, 38), CD132 was constitutively expressed by naïve CD8 T cells (**Figure 3C**). In virus-specific CD8 T cells during chronic infection, CD132 tended to be more abundant in Tpex cells than in the Ttrex and Ttex subsets from both the spleen and bone marrow.

**Figure 4:**
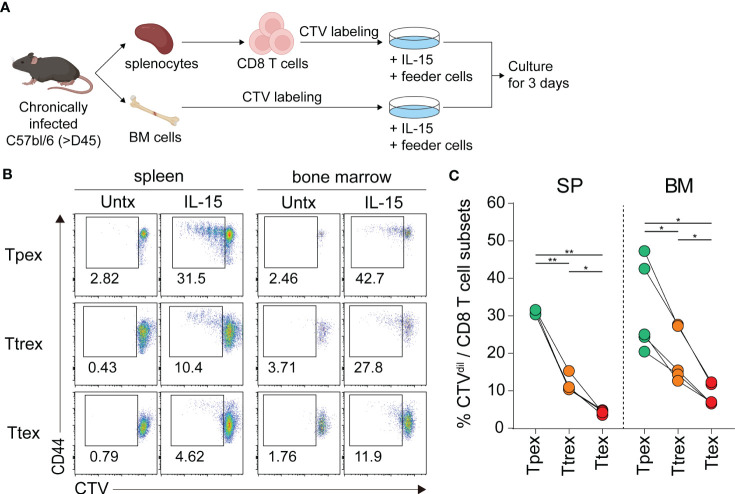
*Ex vivo* IL-15 treatment preferentially induces the proliferation of virus-specific Tpex cells compared with the terminally differentiated subsets. **(A)** Experimental setup: CD8 T cells purified from the spleen and total lymphocytes isolated from the bone marrow of chronically LCMV-infected mice (>day 45 p.i.) were labeled with CTV and cultured with and without recombinant murine IL-15 (100 ng/ml) in the presence of naïve splenocytes used as feeder cells for 3 days. **(B, C)** Representative flow plots **(B)** and a summary graph **(C)** showing the frequency of the Tpex (Tim-3-CD101-), Ttrex (Tim-3+CD101-), and Ttex (Tim-3+CD101+) subsets diluting the CTV among the PD-1+ CD8 T cells from chronically infected mice after 3 days of *ex vivo* culture. Cells from 6 mice per experiment were pooled and cultured in duplicate or triplicate per group per experiment. Data were combined from two independent experiments. *P < 0.05; **P < 0.01 (one-way ANOVA). Statistical significance was determined for the comparison between three subsets in each tissue.

To determine the responsiveness of exhausted CD8 T cell subsets to IL-15 *ex vivo*, we isolated CD8 T cells from the spleen and total lymphocytes from the bone marrow of chronically LCMV-infected mice, labeled them with CTV, cultured them with and without IL-15 for 3 days, and assessed their proliferation (**Figure 4A**). In the absence of antigen stimulation and without any treatment, all three subsets of exhausted CD8 T cells from the spleen and bone marrow had minimal to no proliferation (**Figure 4B**). However, *ex vivo* treatment with IL-15 resulted in a significantly higher proportion of Tpex cells diluting the CTV, and IL-15-driven proliferation gradually decreased in the more terminally differentiated virus-specific CD8 T cell subsets (**Figures 4B, C**). Approximately 30% of Tpex cells from the spleen proliferated upon the addition of IL-15, whereas ~10% of Ttrex and ~5% of Ttex cells divided. This superior proliferative capacity of Tpex cells in response to IL-15, compared with the other subsets, was also observed in cells from the bone marrow. However, the IL-15-induced proliferation levels in Tpex cells were still lower than those in PD-1- CD8 T cells in the same host (**Supplemental Figures 1A, B**) and those in virus-specific memory CD8 T cells generated after acute LCMV infection (**Supplemental Figure 1C**), suggesting that Tpex cells have a compromised ability to undergo proliferation in response to IL-15, compared with naïve or functional memory CD8 T cells. Nonetheless, they possess the highest proliferative capacity in response to IL-15 of all the exhausted CD8 T cell subpopulations.

To exclude any possibilities of the transition of exhausted CD8 T cell subsets during stimulation and directly examine the proliferation of individual T cell subsets, we conducted a similar *ex vivo* experiment after isolating the subsets. We sorted CD73+Tim-3- Tpex and CD73-Tim-3+ Ttex cells based on their exclusive expression of CD73 and Tim-3, respectively (3), from the CTV-labeled splenocytes prepared from chronically LCMV-infected mice. The sorted cells were cultured with or without IL-15 for 3 days, as shown in **Supplemental Figure 2A**. Although the frequency of CTV-diluted Tpex cells was lower than that from the experiment using total CD8 T cells, which might be due to the physical and metabolic stress they underwent during the sorting process, we consistently found that only sorted Tpex cells could proliferate upon *ex vivo* IL-15 treatment, and they preserved a Tim-3-negative phenotype after proliferation (**Supplemental Figures 2B, C**). In contrast, minimal proliferation of sorted Ttex cells was observed after IL-15 treatment and they also retained their Tim-3-positive phenotype. Taken together, these results indicate that *ex vivo* IL-15 treatment preferentially promoted the self-renewal of Tpex cells.

### *Ex vivo* treatment of IL-15 increases the expression of ribosome-related genes but decreases the expression of genes associated with the TCR signaling pathway

**Figure 5:**
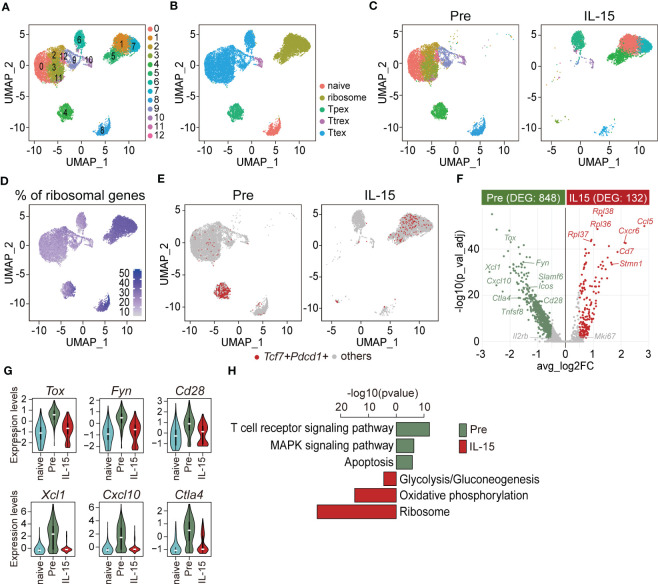
*Ex vivo* IL-15 treatment increases the expression of ribosome-related genes but decreases the expression of genes associated with the TCR signaling pathway in Tpex cells. ScRNA-seq was performed using PD-1+(GP33+GP276)+ CD8 T cells isolated from the spleens of chronically LCMV-infected mice (>day 45 p.i.) before and after *ex vivo* IL-15 treatment for 3 days. Naïve PD-1-CD44^lo^CD62L+ CD8 T cells isolated from the spleens of naïve mice were included as a control. **(A, B)** UMAP plots showing unsupervised Seurat clusters **(A)** and supervised cluster groupings based on the phenotypes **(B)** of tetramer+ CD8 T cells. **(C)** Distribution of pre- (left) and post-IL-15 treatment (right, IL-15) samples on unsupervised Seurat clusters. **(D)** Expression of ribosomal genes across integrated samples. **(E)** Distribution of *Tcf7+Pdcd1+* cells of pre- (left) and post-IL-15 treatment (right) samples. **(F)** Comparison of DEGs between pre- and post-IL-15 treatment samples of *Tcf7*+*Pdcd1*+ cells. The volcano plot shows the average fold-change (log2) versus the adjusted P-value (-log10) for individual genes. Significance was determined as |log2fold-change > log2(0.5)| and adjusted P-value < 0.05 (two-sided Wilcoxon test). The number of DEGs is shown in the subtitles. **(G)** Violin plots of representative genes highly expressed in the pre-treatment *Tcf7+Pdcd1+* cells. **(H)** KEGG pathway analysis of the DEGs between pre- and post-IL-15 treatment samples of *Tcf7*+*Pdcd1*+ cells. A false discovery rate (FDR) < 0.05 indicates a significant change. Data are representative of 15 biologically independent pooled samples.

To understand the IL-15-driven alterations of exhausted CD8 T cells in greater detail, we isolated GP33- and GP276-specific CD8 T cells from chronically LCMV-infected mice (>45 days p.i.) before and after 3-days of *ex vivo* IL-15 stimulation and performed scRNA-seq for transcriptomic analysis. Naïve PD-1-CD44^lo^CD62L+ CD8 T cells isolated from naïve mice were included as a control for scRNA-seq analysis. Sub-clustering of the LCMV-specific exhausted CD8 T cells identified 13 clusters (**Figure 5A**), and they were functionally annotated as shown in **Figure 5B**. As previously described (11, 39), we confirmed that LCMV-specific CD8 T cells in chronically-infected mice consisted of Tpex, Ttrex, and Ttex cells (**Figure 5C**, left [pre]). Of interest, *ex vivo* IL-15 treatment resulted in a large change in the uniform manifold approximation and projection (UMAP) distribution of tetramer-positive exhausted CD8 T cells and this alteration was largely dependent on the expression of ribosomal genes (**Figure 5C**, right [IL-15], **Figure 5D**). We first focused on the adjustment of Tpex cells. Upon ex *vivo* IL-15 treatment, *Tcf7+Pdcd1+* Tpex cells moved from cluster 4 to clusters 1, 5, and 7 (**Figure 5E**). The pair-wise analysis demonstrated that IL-15 treatment increased the expression of genes encoding ribosomal proteins such as *Rpl36*, *Rpl37*, and *Rpl38*, the naïve/memory marker *Cd7*, the proliferation marker *Stmn1*, and chemokine/chemokine receptors *Ccl5* and *Cxcr6* in Tpex cells (**Figure 5F**). In contrast, the expression levels of the Tpex-defining marker *Slamf6*, co-stimulatory molecules *Cd28*, *Icos*, and *Tnfsf8*, the inhibitory receptor *Ctla4*, the transcription factor *Tox*, chemokines *Xcl1* and *Cxcl10*, and the TCR signaling molecule *Fyn* were significantly decreased in Tpex cells by the treatment. We found that the levels of *Tox*, *Fyn*, and *Cd28* were still higher in Tpex cells after IL-15 treatment than those in naïve CD8 T cells, while *Xcl1*, *Cxcl10*, and *Ctla4* levels of Tpex cells were decreased to a level which was similar to those from naïve CD8 T cells (**Figure 5G**). KEGG pathway analysis revealed that gene sets enriched in Tpex cells after the IL-15 treatment were mostly associated with the ribosome in addition to oxidative phosphorylation and glycolysis/gluconeogenesis (**Figure 5H**). In contrast, the expression of genes related to the TCR pathway, the mitogen-activated protein kinase (MAPK) signaling pathway, and apoptosis was reduced in Tpex cells following the treatment.

We next compared the transcriptome of Ttex cells before and after IL-15 treatment. Prior to treatment, Ttex cells primarily resided in clusters 0, 2, 3, 9, 10, 11, and 12, while after treatment, they were predominantly located in clusters 1, 5, 6, and 7 (**Supplemental Figure 3A**). The pair-wise analysis revealed that the list of DEGs in Ttex cells was quite similar to that of Tpex cells. For example, the expression of ribosomal proteins such as *Rpl22*, *Rpl36*, *Rpl37*, *Rpl38*, *Rpl39*, and *Rpl41* and the anti-apoptotic molecule *Bcl2* in addition to *Stmn1* was augmented in Ttex cells after IL-15 treatment (**Supplemental Figure 3B**). Conversely, pre-treatment Ttex cells exhibited higher expression of inhibitory receptors *Ctla4* and *Lag3*, the co-stimulatory molecule *Tnfrsf9*, the effector cytokine *Ifng*, the cytolytic molecule *Gzma*, chemokines *Ccl3* and *Ccl4*, and *Il2rb* in addition to *Tox* and *Fyn*. We also found that the *Tox*, *Fyn*, and *Gzma* levels of Ttex cells were still higher after IL-15 treatment than those of naïve CD8 T cells, while their levels of *Ccl3*, *Lag3*, and *Ctla4* after the treatment were reduced to a level comparable to that of naïve CD8 T cells (**Supplemental Figure 3C**). Additionally, the expression of ribosome-, glycolysis/gluconeogenesis-, and biosynthesis of amino acids-related genes was up-regulated, while the expression of genes associated with the TCR signaling pathway, apoptosis, and the MAPK signaling pathway was reduced in Ttex cells after IL-15 treatment (**Supplemental Figure 3D**). Taken together, these results suggest that *ex vivo* IL-15 treatment promoted the translational machinery of exhausted CD8 T cells and anti-apoptotic mechanisms similarly in both Tpex and Ttex subsets. On the other hand, the TCR signaling pathway was mitigated in both subsets upon IL-15 treatment compared with the pre-treatment cells, which might be associated with the 3-day *ex vivo* culture without antigens.

### *In vivo* administration of IL-15 promotes the self-renewal of Tpex cells during chronic LCMV infection

**Figure 6:**
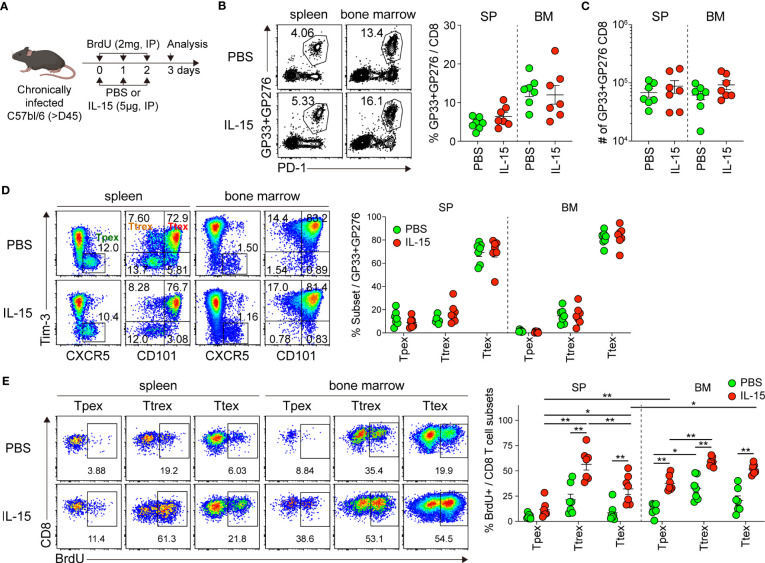
*In vivo* administration of IL-15 promotes the self-renewal of Tpex cells during chronic LCMV infection. **(A)** Experimental setup: Chronically LCMV-infected mice (>day 45 p.i.) were treated with either PBS or IL-15 (5 µg) together with BrdU (2 mg) *via* daily i.p. injections for 3 days. Analysis was performed one day after the last treatment. **(B, C)** Frequency of GP33+GP276-specific CD8 T cells **(B)** and their absolute numbers **(C)** in the spleen and bone marrow. **(D)** Frequency of the Tpex (CXCR5+Tim-3-), Ttrex (Tim-3+CD101-), and Ttex (Tim-3+CD101+) subsets among GP33+GP276-specific CD8 T cells in the spleen and bone marrow. **(E)** Frequency of each subset of GP33+GP276-specific CD8 T cells that incorporated BrdU in the spleen and bone marrow. Data were combined from two independent experiments with n=3–4 mice per group per experiment. The graphs show the mean and SEM. *P < 0.05; **P < 0.01 (one-way ANOVA). Statistical significance was determined only in the following cases: 1) comparison between PBS and IL-15 treatment for each subset, 2) comparison among three subsets per treatment in each tissue, and 3) comparison between the spleen and bone marrow for the corresponding subsets.

To test the proliferation of exhausted CD8 T cells in response to IL-15 *in vivo*, chronically LCMV-infected mice were treated daily with either PBS or IL-15 together with BrdU for 3 days and then sacrificed the day after the last treatment (**Figure 6A**). Short-term IL-15 administration did not significantly alter the frequency or number of GP33+GP276-specific CD8 T cells in the spleen or bone marrow (**Figures 6B, C**). We also found that the magnitude of LCMV-specific CD8 T cell responses was similar in blood pre- and post-IL-15 treatment (**Supplemental Figure 4**). Among the GP33+GP276-specific CD8 T cells in tissues, the proportions of the Tpex, Ttrex, and Ttex subsets were comparable between mice that received PBS and those that received IL-15 (**Figure 6D**). This result is different from that after IL-2 treatment, which generated a distinct effector-like subset of CD8 T cells during chronic LCMV infection (39).

When assessing BrdU uptake, consistent with our result from the *ex vivo* IL-15 treatment (**Figure 4**), we found that the frequency of BrdU+ cells among the Tpex cells from the spleen and bone marrow was significantly higher in the IL-15-treated mice than in the mice receiving PBS (**Figure 6E**). Moreover, it is noteworthy that the magnitude of IL-15-driven homeostatic proliferation of Tpex was significantly higher in the bone marrow than in the spleen. However, the BrdU+ proportion of the Tpex subset was still lower than that of PD-1- CD8 T cells in the same mouse (**Supplemental Figures 5A, B**), as we observed after *ex vivo* IL-15 treatment. On the other hand, different from our results in the *ex vivo* culture with IL-15, substantially increased proliferation occurred in the Ttrex and Ttex subsets after *in vivo* IL-15 treatment. In sum, these data show that IL-15 promotes the self-renewal of Tpex cells *in vivo*, supporting their maintenance during chronic viral infection.

### Responsiveness of CD8 TILs in human RCC to *ex vivo* IL-15 treatment

**Figure 7:**
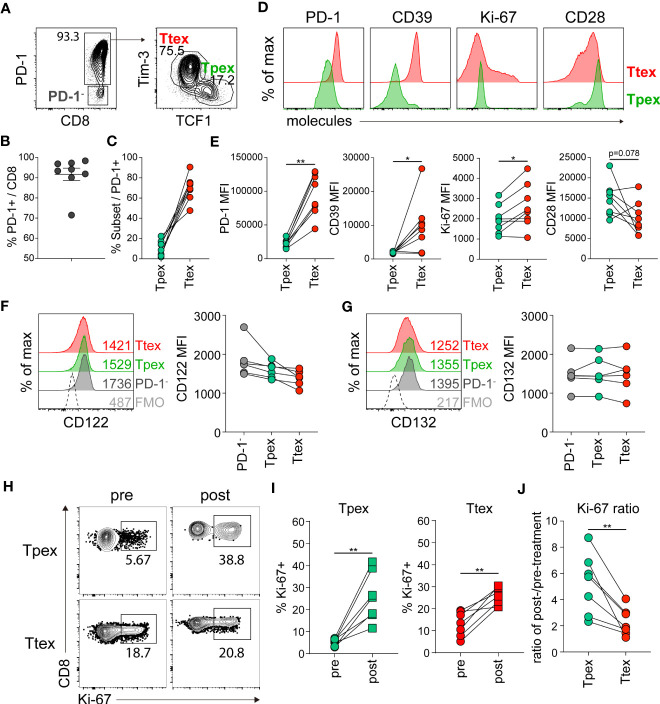
Tumor-infiltrating Tpex cells from human RCC have the superior proliferative capacity in response to *ex vivo* IL-15 treatment compared with the Ttex subset of CD8 TILs. **(A)** Representative flow plots of PD-1 expression on CD8 TILs (left) and the frequency of TCF1+Tim-3- Tpex and TCF1-Tim-3+ Ttex cells among PD-1+ CD8 TILs (right) in human RCC tumors. **(B)** Frequency of PD-1+ cells among total CD8 TILs. The graph shows the mean and SEM. **(C)** Frequency of Tpex and Ttex cells among PD-1+ CD8 TILs. **(D, E)** Representative flow plots **(D)** and summary graphs **(E)** showing the expression of PD-1, CD39, Ki-67, and CD28 on Tpex and Ttex cells. Data were combined from three independent experiments with samples from 8 patients. *P < 0.05; **P < 0.01 (Student’s *t*-test, paired). **(F, G)** Expression of CD122 **(F)** and CD132 **(G)** on Tpex and Ttex cells among PD-1+ CD8 TILs. Data were combined from two independent experiments with samples from 6 patients. No statistical significance was determined by one-way ANOVA. **(H-J)** RCC TILs were cultured with recombinant human IL-15 (10 ng/ml) in the presence of irradiated autologous PBMCs for 3 days. **(H, I)** Frequency of Ki-67+ cells in the Tpex and Ttex subsets pre- and post-treatment. **(J)** The expansion ratio from pre- to post-treatment in each subset. Data were combined from two independent experiments with samples from 7 patients. **P < 0.01 (Student’s *t*-test, paired).

Next, we asked whether IL-15 could also influence the proliferation of exhausted CD8 T cells in a human tumor microenvironment. In this study, we investigated CD8 TILs obtained from tumors resected from RCC patients. Our findings revealed that PD-1 was expressed in approximately 91% of tumor-infiltrating CD8 T cells, with a range of 71.5 to 98.4% (**Figure 7A, B**). As previously described (8), we also found distinct populations of TCF1+Tim-3- Tpex and TCF1-Tim-3+ Ttex cells among the PD-1+ CD8 TILs (**Figures 7A, C**), and they exhibited phenotypic characteristics similar to the corresponding exhausted CD8 T cell subsets detected in chronic LCMV infection (3) (**Figures 7D, E**). The Tpex cells expressed a marginally higher level of CD28 and an intermediate level of PD-1 and did not express CD39, whereas the Ttex cells expressed CD39 and higher levels of PD-1 and Ki-67, indicating that the Tpex subset of CD8 TILs was less exhausted than the Ttex cells. However, in the 19 RCC patients we examined, 5 individuals (26.3%) had no Tim-3^hi^ population of PD-1+ CD8 TILs. In those individuals, the PD-1+ CD8 TILs included TCF1- cells with low Tim-3 and Ki-67 expression and variable amounts of CD39, as well as TCF1+ cells expressing elevated CD28 and intermediate PD-1 levels (**Supplemental Figure 6**).

We next compared the expression of CD122 and CD132 in human CD8 TIL subsets. In 8 cases in which we assessed IL-15 receptor expression, almost no detectable Tim-3^hi^ population of PD-1+ CD8 T cells was found in 2 of them. Therefore, we analyzed the expression of IL-15 receptors in the TCF1+Tim-3- Tpex and TCF1-Tim-3+ Ttex subsets from the 6 patients who had Tim-3^hi^PD-1+ CD8 T cells. In contrast to their expression in chronically LCMV-infected mice, the expression of CD122 and CD132 was similar among PD-1- CD8 T cells, Tpex, and Ttex cells (**Figures 7F, G**).

To examine whether exhausted CD8 TIL populations from human tumors respond to IL-15, we cultured RCC TILs *ex vivo* in the presence of IL-15 for 3 days and evaluated proliferation by analyzing Ki-67 expression. All patients in this experiment (n=7) had Tim-3^hi^PD-1+ cells among their CD8 TILs. We found that *ex vivo* IL-15 treatment significantly increased the percentage of Ki-67+ cells in both the Tpex and Ttex subsets, compared with the pre-treatment levels (**Figures 7H, I**). Because we did not isolate CD8 T cells for the *ex vivo* culture, it is likely that APCs harboring antigens might have caused antigen-driven proliferation. Of interest, when we compared the expansion ratio from pre- to post-treatment, the Ttex subset exhibited a 1- to 4-fold increase in the IL-15-driven proliferation condition, whereas the magnitude of the increase in Tpex proliferation caused by IL-15 treatment was much higher, 2- to 9-fold (**Figure 7J**). However, it is noteworthy that the IL-15-driven self-renewal of the Tpex subset was still lower than that of PD-1- CD8 TILs (**Supplemental Figure 7**), suggesting the compromised proliferative ability of Tpex cells. In conclusion, although each individual presented variable IL-15-driven expansion ratios of the Tpex subset from pre- to post-treatment, suggesting a differential quality among patients, these results indicate that IL-15 could also preferentially enhance the self-renewal of the Tpex subset of human CD8 TILs.

## Discussion

In this study, although the number of Tpex cells was significantly reduced one-year p.i., we observed self-renewal of Tpex cells in the lymphoid organs, primarily in the bone marrow, during chronic viral infection. Interestingly, we also found that *ex vivo* and *in vivo* treatments of recombinant IL-15 could induce homeostatic proliferation of Tpex cells in both a murine model of chronic viral infection and human cancer. Furthermore, *ex vivo* IL-15 treatment upregulated the expression of ribosome-related genes and downregulated the expression of genes associated with the TCR signaling pathway and apoptosis in both the Tpex and Ttex subsets.

It has been thought that CD122 expression is associated with responsiveness to IL-15. As previously observed (36, 40), we confirmed that the central memory subset of virus-specific CD8 T cells, which were generated following LCMV Armstrong infection and have higher CD122 expression, exhibited better proliferative capacity in response to *ex vivo* IL-15 treatment than the effector memory subset (data not shown). However, in human RCC TILs, we found that CD122 expression was comparable between Tpex and Ttex cells. Moreover, in chronic LCMV infection, higher expression of CD122 was shown on the terminally differentiated subsets than on Tpex cells, which does not reflect their responsiveness to IL-15 but is consistent with other reports that higher levels of CD122 were found on the more exhausted LCMV-specific or hepatitis C virus–specific CD8 T cells (39, 41). Similarly, there was a trend toward lower CD122 expression on CD28+ cells that co-expressed TCF1 than on CD28- cells, which were considered to be terminally differentiated cells, among the PD-1+ CD8 TILs from human non-small-cell lung cancer (42). On the other hand, CD122 was expressed at similar levels on TCF1+ and TCF1- cells among OVA-specific CD8 TILs in the murine MC38-OVA model in that same study. Thus, no common expression pattern of CD122 was found among the exhausted CD8 T cell subsets during chronic viral infection and cancer. It seems that CD122 expression by exhausted CD8 T cells alone does not entirely correlate with the responsiveness to IL-15, which could instead be determined by other CD8 T cell subset-intrinsic and -extrinsic factors, as well as IL-15 receptor expression in the context of persistent antigenic stimulation. It would also be of interest to investigate whether the CD122 and CD132 molecules that we examined exist in the receptor complex or in a free form. Further examinations of that issue are required.

IL-15 belongs to the common γ chain family of cytokines consisting of six members (IL-2, IL-4, IL-7, IL-9, IL-15, and IL-21) that all have unique receptors for specificity and exert distinct functions that influence the differentiation, proliferation, and effector behavior of T cells (43). A recent study showed that only Tpex cells responded and proliferated in response to *in vivo* IL-2 treatment during chronic LCMV infection, generating transcriptionally and epigenetically different effector-like cells, whereas terminally differentiated cells exhibited minimal to no expansion upon IL-2 treatment, indicating that among virus-specific exhausted CD8 T cells, only Tpex cells exhibit responsiveness to IL-2 (39). IL-7, like IL-15, plays a crucial role in the survival and maintenance of memory CD8 T cells. During chronic LCMV infection, Tpex cells were found to express higher levels of CD127 (IL-7Rα) compared to terminally differentiated cells (3). Additionally, a small population of exhausted CD8 T cells was observed to undergo division in response to *ex vivo* IL-7 treatment (14). Furthermore, in chronic hepatitis C virus infection, it has been shown that a PD-1+CD127+TCF1+ progenitor subset with elevated levels of CD127 was maintained after removal of antigen stimulation and generated more exhausted PD-1^hi^CD127-TCF1- CD8 T cells upon antigenic stimulation (44, 45). These findings suggest that IL-7 might have a major effect on Tpex cells and support the need for further study. IL-21 was previously shown to play a critical role in the maintenance of exhausted CD8 T cells and the control of chronic viral infections (46–48). Recently, the specific role of IL-21 was revealed in a study in which the depletion of CD4 T cells or IL-21R-deficient virus-specific CD8 T cells significantly impaired the development of CX3C chemokine receptor 1 (CX3CR1)+ Ttrex cells during chronic LCMV infection (49), indicating that IL-21 produced from CD4 T cells is required for differentiation into Ttrex cells, likely by promoting differentiation from Tpex cells. In addition, transferring IL-21-expressing CD4 T cells into B16F10 tumor-bearing mice enhanced the proliferation of CX3C chemokine receptor 1+ CD8 TILs, which correlated with improved tumor control. Therefore, common γ chain cytokines seem to act primarily on the Tpex subset, but they have different influences on its proliferation and differentiation, and that has implications for the therapeutic manipulation of those cytokines to improve T cell–based cancer immunotherapies.

In preclinical and clinical studies, IL-15 has been evaluated as a potential treatment against cancer as a monotherapy and in combination with other immunotherapeutic agents. IL-15 administration combined with immune checkpoint inhibitors targeting PD-1/programmed cell death ligand-1 has been shown to improve tumor control and survival compared with either treatment alone in multiple murine tumor models (42, 50–52). Currently, several clinical trials examining the combination of recombinant human IL-15 or IL-15 agonists with nivolumab and/or ipilimumab are ongoing in multiple types of advanced or refractory cancers (NCT02523469, NCT03228667, NCT03520686, and NCT033886320). In a recent phase Ib clinical trial, subcutaneous administration of 20 μg/kg of an ALT-803, IL-15/IL-15Rα complex fused to IgG1 Fc, in combination with nivolumab every 2 weeks in metastatic non-small-cell lung cancer patients produced an objective response of 29%, with 6 of 21 patients experiencing a partial response and 16 of 21 (76%) patients achieving disease control (53). Of note, 10 of 11 (91%) patients who had progressed after previous αPD-1 therapy manifested disease control, with 3 individuals (27%) showing a partial response, suggesting that the provision of IL-15 could overcome resistance to αPD-1 treatment. These preclinical and clinical studies suggest that IL-15 co-delivery augments the proliferation of CD8 T cells and NK cells (17, 51–53) and elevates the expression of granzyme B and IFNγ by CD44^hi^ CD8 T cells at the site of metastases and the expression of inflammatory cytokines such as IFNγ and TNF (51). Although the induction of inflammatory cytokines or augmented NK cell activity caused by IL-15 treatment could play a role in its therapeutic efficacy, our results suggest that replenishing Tpex cells could be another mechanism by which combined IL-15 treatment is efficacious. Because PD-1 pathway blockade acts mainly on Tpex cells, causing them to generate Ttrex cells with strong cytolytic activity (11), an administration of IL-15 that increases the population of Tpex cells combined with αPD-1 therapy could lead to a powerful synergistic effect that might be applied to more patients and different types of cancer that are resistant or refractory to current therapies.

In adoptive cell therapy, although more differentiated cells exhibit stronger cytolytic activity *in vitro*, less differentiated CD8 T cells mediate more potent antitumor activity *in vivo* with enhanced persistence (54–56). Consistently, it has been reported that transferring Tpex cells isolated from TILs into B16-OVA-bearing mice controlled tumor growth significantly better than transferring Ttex cells (9). Moreover, in the same study, combined therapy of αPD-1 antibody treatment specifically increased the number of transferred Tpex cells, but not Ttex cells, implying the importance of the Tpex subset in the context of adoptive T cell therapy as well. However, in adoptive T cell therapy using chimeric antigen receptor (CAR) T cells or TILs, the *ex vivo* expansion procedure used in the clinic involves T cell receptor stimulation together with IL-2, which is more likely to induce terminal differentiation, limiting *in vivo* persistence and durable therapeutic efficacy (57). Instead, according to our results, IL-15 preferentially increases the Tpex population, limiting further differentiation. Analogous to these results, CAR T cells cultured in IL-7/IL-15 generated more T_CM_-like cells and showed an improved response to αPD-1 therapy following transfer into tumor-bearing mice, compared with cells preconditioned in IL-2/IL-15 (58). Similarly, CAR T cells targeting vascular endothelial cell growth factor receptor 2 cultured in IL-2 followed by IL-7/IL-15 showed enhanced viability and expansion, generating a higher proportion of T_CM_ cells and also increased expression of effector molecules and improved persistence, when they were co-cultured with target antigen-expressing cells, compared with those cultured in IL-2 alone (59). Therefore, CAR T cells engineered to co-express IL-15 produced better tumor control in a mouse model. Because a high proportion of Tpex cells or less differentiated cells in the infusion product correlates with better persistence and antitumor activity *in vivo*, the addition of IL-15, which increases Tpex cells without further differentiation, could be applied to the *ex vivo* expansion protocol for adoptive T cell therapy for cancer patients.

It has been shown in melanoma patients that responders to αPD-1 therapy possessed more TCF1+ CD8 T cells in their tumors than non-responders (13). In an independent study of melanoma patients, the frequency of TCF1+PD-1+ CD8 TILs did not predict the responsiveness to immune checkpoint blockades, which was different from an earlier study, but a higher percentage of Tpex cells among PD-1+ CD8 TILs was associated with prolonged progression-free survival in responders (9). In our data, the Tpex subset of CD8 TILs in RCC proliferated in response to IL-15 better than the Ttex cells in all individuals examined, but the level of expansion induced by IL-15 varied among patients, implying that Tpex cells have differential functionality in each individual. Considering the exclusive proliferative potential of Tpex cells upon PD-1 pathway blockade (3–5, 9, 10, 12), it is of interest to further investigate whether the magnitude of proliferation in Tpex cells upon IL-15 treatment could be used to predict the response to immune checkpoint blockade.

This study has a few limitations. Although we found IL-15-driven self-renewal of Tpex cells upon ex *vivo* and *in vivo* IL-15 treatment as a proof of concept, it is still questionable how long Tpex cells could proliferate after the continuous treatment and how the functional features of Tpex cells would change after prolonged IL-15 treatment. It would be valuable to determine optimal therapeutic regimens for combination with αPD-1 therapy and for the expansion of CD8 T cells without further differentiation for the adoptive T cell therapy. Next, to explain the discordant results of IL-15-driven Ttex proliferation between *ex vivo* and *in vivo* experiments, we attempted an *in vivo* experiment using sorted Tpex and Ttex cells, but we were unable to observe IL-15-driven homeostatic proliferation in either subset of transferred cells (data not shown), which might be due to the significant physical and metabolic stress during the sorting process. Additionally, transferred cells might settle down in a different location from their original sites, where optimal IL-15 presentation does not occur. Considering the limited proliferative potential of Ttex cells upon antigenic rechallenge or the treatments with αPD-1 antibodies and recombinant IL-2 (3, 39), and our current data in which Ttex cells minimally proliferate after *ex vivo* IL-15 stimulation, it is likely that the increased proliferation of Ttrex and Ttex subsets following *in vivo* IL-15 treatment mainly results from the transition of Tpex cells rather than from their self-renewal. However, we would not completely exclude other possibilities that the different interactions or signaling pathways by IL-15 *in vivo* could also affect their proliferation. Lastly, in the experiment of *ex vivo* IL-15 treatment with human RCC samples, we compared proliferation between pre- and post-treatment without including a media control group because we conducted other phenotypic analyses simultaneously with the limited number of cells obtained. Therefore, the interpretation of the results should be made carefully, taking the 3-day incubation period without antigens into consideration.

In summary, we found that Tpex cells self-renew in the spleen and (predominantly) bone marrow during chronic viral infection, but their population gradually declines during the course of infection. Furthermore, *ex vivo* and *in vivo* treatment with IL-15 preferentially increased the proliferation of Tpex cells from chronically infected mice, although their proliferative capability in the presence of IL-15 was still lower than the PD-1- population in the same mice or virus-specific memory CD8 T cells. In addition, tumor-infiltrating Tpex cells from human RCC expanded in response to IL-15 better than the Ttex subset of CD8 TILs. Our finding that the homeostatic proliferation of Tpex cells occurs in lymphoid tissues and that IL-15 can promote the self-renewal of Tpex cells *ex vivo* and *in vivo* offers a new insight into the maintenance of Tpex cells and has important therapeutic implications.

## Data availability statement

The original contributions presented in the study are publicly available. This data can be found here: [https://www.ncbi.nlm.nih.gov/geo/query/acc.cgi?acc=GSE233388].

## Ethics statement

The studies involving human participants were reviewed and approved by the Institutional Review Board at Samsung Medical Center. The patients/participants provided their written informed consent to participate in this study. The animal study was reviewed and approved by Institutional Animal Care and Use Committee guidelines of the Sungkyunkwan University School of Medicine.

## Author contributions

SI, JL, and KyL designed and performed the experiments and analyzed data. MK provided the patient samples. KyL, HB, KuL, SL, JM, and KJ participated in sample preparation. IK and BJ analyzed data. SI, JL, and KyL wrote the manuscript. SI supervised the overall study. All authors contributed to the article and approved the submitted version.
