# Supplementary material for: IL-15 promotes self-renewal of progenitor exhausted CD8 T cells during persistent antigenic stimulation
Source: Front Immunol. 2023 Jun 20;14:1117092. doi: 10.3389/fimmu.2023.1117092 (PMC10319055; doi:10.3389/fimmu.2023.1117092)
Supplement: Supplementary file 1 [file DataSheet_1.pdf]

Supplementary Figure 1. Lee JH, Lee KM, and et al.

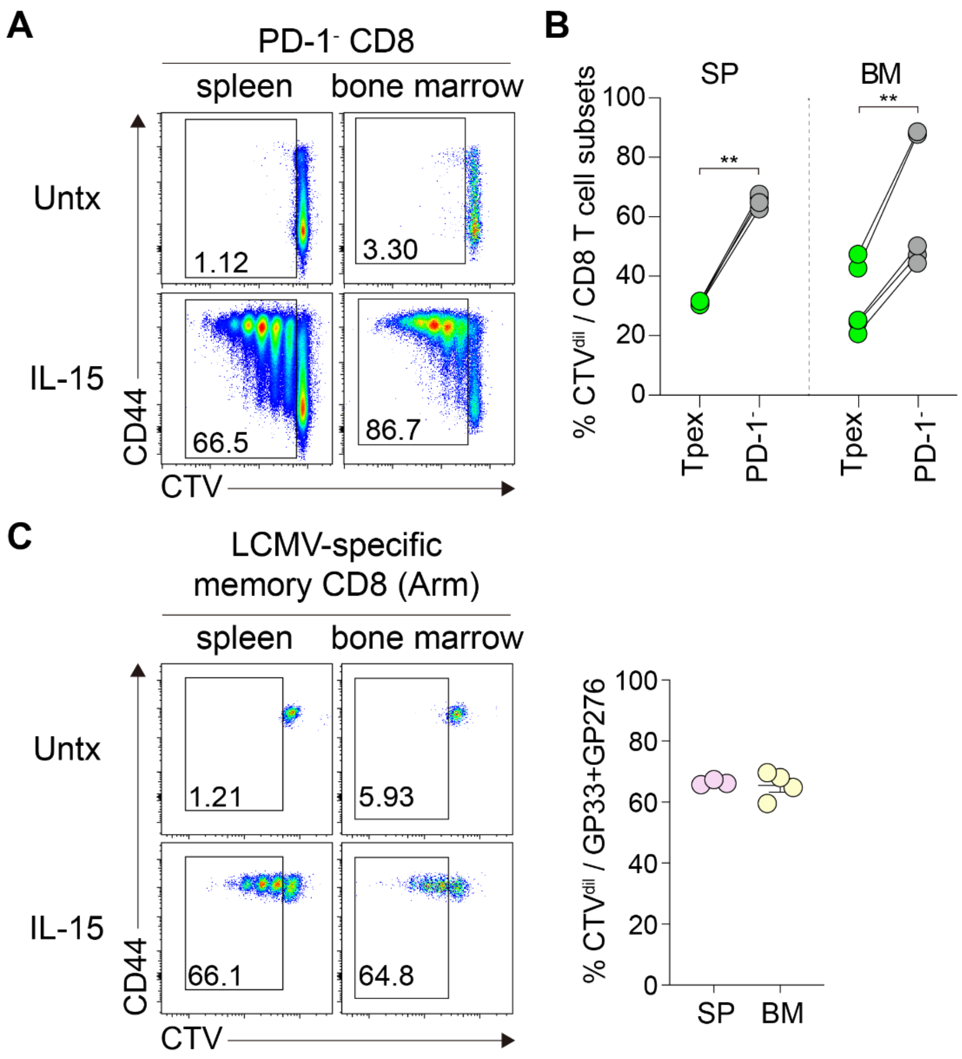

Supplemental Figure 1. Tpex cells have compromised proliferative capacity in response to *ex vivo* treatment with IL-15 compared with naïve CD8 T cells or virus-specific memory CD8 T cells. The same experimental setup as described in Figure 4 was used. (**A**) Representative flow plots showing the proliferation of PD-1- CD8 T cells isolated from chronically LCMV-infected mice. (**B**) Comparison of IL-15-driven proliferation between Tpex cells (which is shown in Figure 4B, C) and PD-1- CD8 T cells from the same host after 3 days of *ex vivo* culture with IL-15. Cells were pooled from 6 mice per experiment and cultured in duplicate or triplicate per group per experiment. Data were combined from two independent experiments. **$P < 0.01$ (Student's *t*-test, paired). (**C**) Frequency of CTV-diluted GP33+GP276-specific memory CD8 T cells after 3 days of *ex vivo* culture. CD8 T cells and total lymphocytes were purified from the spleens and bone marrow, respectively, of mice with cleared LCMV Armstrong infections (>day 90 p.i.), labeled with CTV, and cultured with or without recombinant murine IL-15 (100ng/ml) in the presence of naïve splenocytes as feeder cells for 3 days. Cells were pooled from 3–6 mice per experiment and cultured in triplicate or quadruplicate per group per experiment. Data are representative of three independent experiments. The graph shows the mean and SEM.

Supplementary Figure 2. Lee JH, Lee KM, and et al.

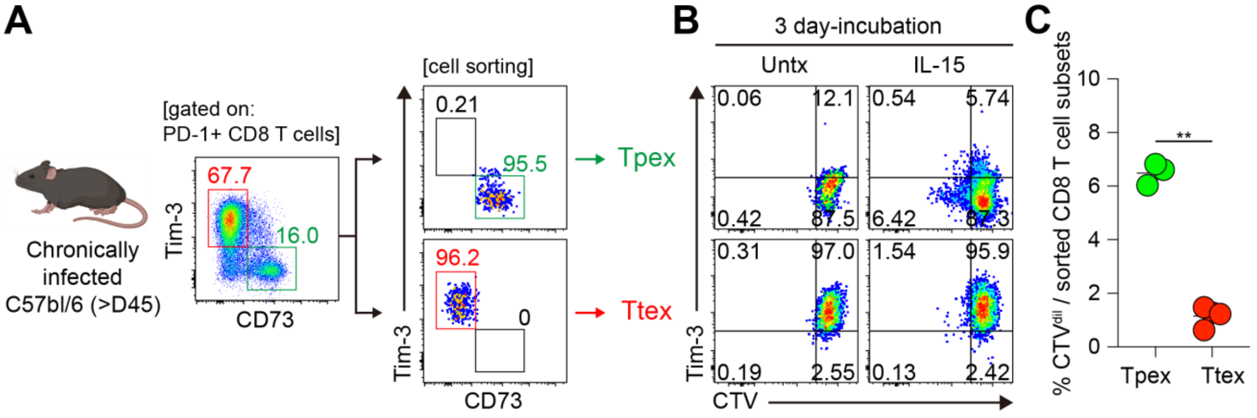

Supplemental Figure 2. ***Ex vivo*** **IL-15 treatment preferentially promotes the proliferation of Tpex cells compared with Ttex cells.** (**A**) Sorting strategy. CD73+Tim-3- Tpex cells and CD73-Tim-3+ Ttex cells among PD-1+ CD8 T cells were isolated from the CTV-labeled splenocytes of chronically LCMV-infected mice (>day 45 p.i.). Sorted cells were cultured with or without recombinant murine IL-15 (100 ng/ml) in the presence of naïve splenocytes for 3 days. (**B, C**) Representative flow plots (**B**) and a summary graph (**C**) showing the frequency of sorted Tpex and Ttex cells diluting the CTV after 3 days of *ex vivo* culture. Cells from 12 mice were pooled for sorting and cultured in triplicate (Tpex) or quadruplicate (Ttex). The graph shows the mean and SEM. $^{**}P < 0.01$ (Student's t-test).

Supplementary Figure 3. Lee JH, Lee KM, and et al.

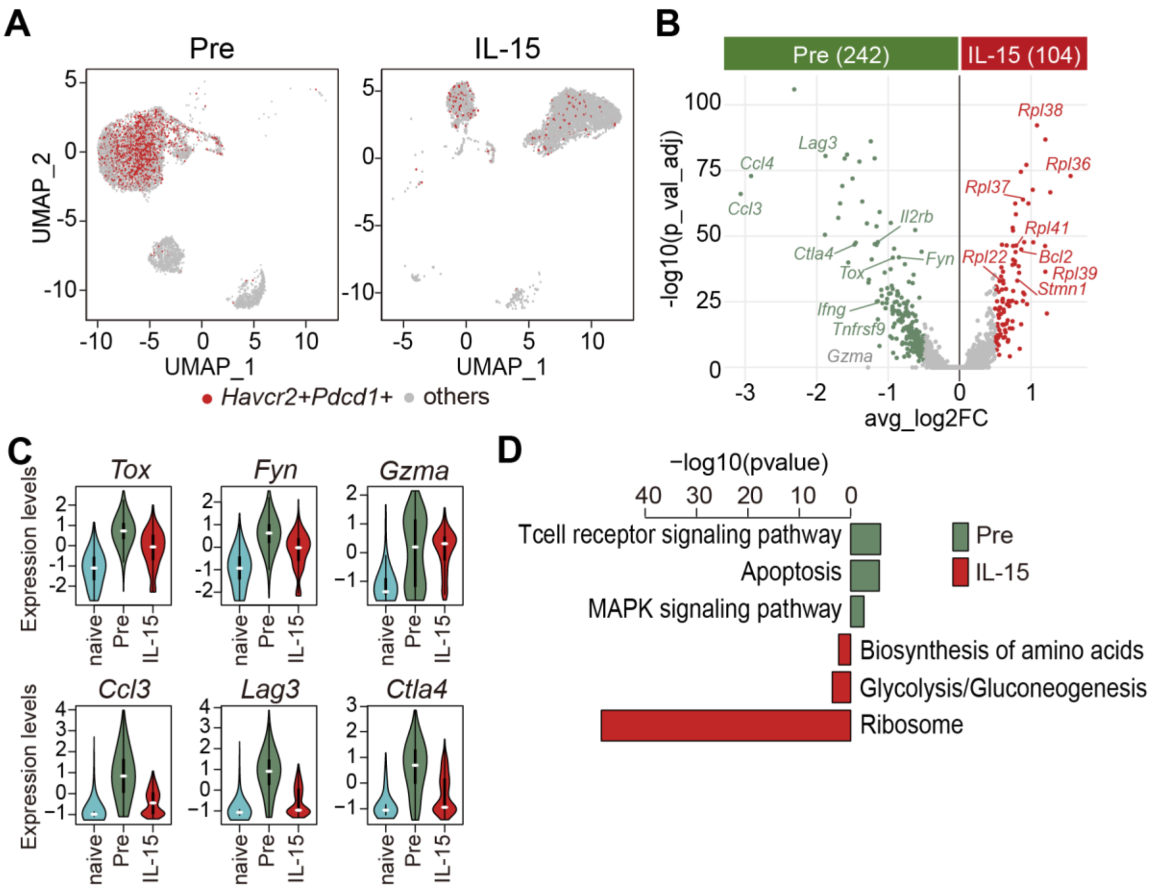

Supplemental Figure 3. ***Ex vivo*** **IL-15 treatment increases the expression of ribosome-related genes but decreases the expression of genes associated with the TCR signaling pathway in Ttex cells.** ScRNA-seq was performed as described in Figure 5. (**A**) Distribution of *Havcr2+Pdcd1+* cells of pre- (left) and post-IL-15 treatment (right, IL-15) samples (**B**) Comparison of DEGs between pre- and post-IL-15 treatment samples of *Havcr2+Pdcd1+* cells. The volcano plot shows the average fold-change (log2) versus the adjusted P-value (-log10) for individual genes. Significance was determined as |log2fold-change > log2(0.5)| and adjusted P-value < 0.05 (two-sided Wilcoxon test). The number of DEGs is shown in the subtitles. (**C**) Violin plots of representative genes highly expressed in the pre-treatment *Havcr2+Pdcd1+* cells. (**D**) KEGG pathway analysis of the DEGs between pre- and post-IL-15 treatment samples of *Havcr2+Pdcd1+* cells. A false discovery rate (FDR) < 0.05 indicates a significant change. Data are representative of 15 biologically independent pooled samples.

Supplementary Figure 4. Lee JH, Lee KM, and et al.

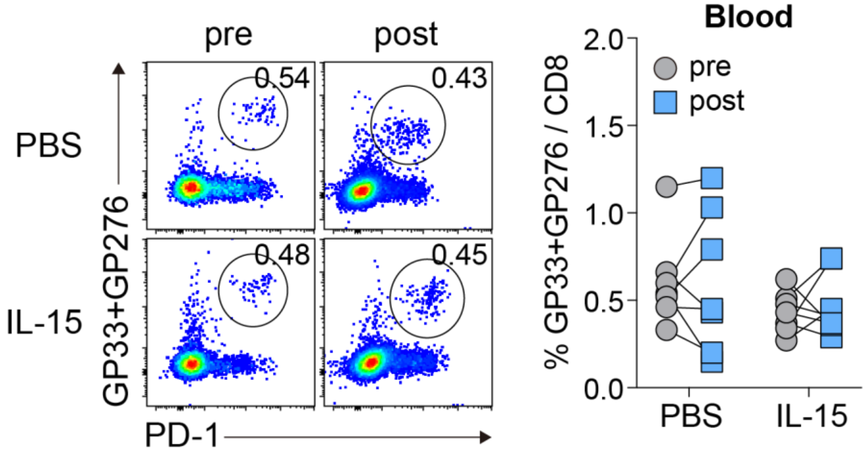

Supplemental Figure 4. Chronically LCMV-infected mice that received exogenous IL-15 show no significant change in the magnitude of virus-specific CD8 T cells in blood. The same experimental setup as described in Figure 6 was used. Representative flow plots and a summary graph showing the frequency of GP33+GP276-specific CD8 T cells in blood pre- and post-treatment. The results were combined from two independent experiments with n=3–4 mice per group per experiment.

Supplementary Figure 5. Lee JH, Lee KM, and et al.

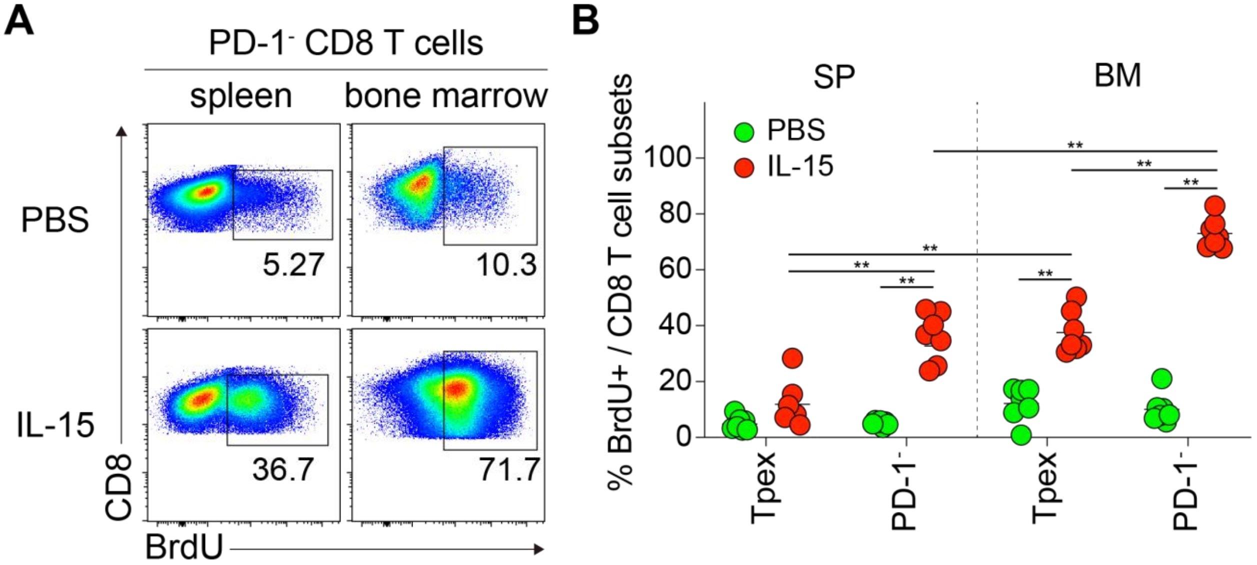

Supplemental Figure 5. Virus-specific Tpex cells have an impaired ability to proliferate upon *in vivo* IL-15 treatment compared with naïve CD8 T cells. The same experimental setup as described in Figure 6 was used. (**A**) Representative flow plots showing the BrdU incorporation of PD-1- CD8 T cells from the spleen and bone marrow of chronically LCMV-infected mice that received PBS or IL-15. (**B**) Comparison of BrdU incorporation following *in vivo* IL-15 administration between GP33+GP276-specific Tpex cells (which is shown in Figure 6E) and PD-1- CD8 T cells from the same host. Data were combined from two independent experiments with n=3–4 mice per group per experiment. The graph shows the mean and SEM. $^{**}P < 0.01$ (one-way ANOVA). Statistical significance was determined only in the following cases: 1) comparison between PBS and IL-15 treatment for each subset, 2) comparison between two subsets per treatment in each tissue, and 3) comparison between the spleen and bone marrow for the corresponding subsets.

Supplementary Figure 6. Lee JH, Lee KM, and et al.

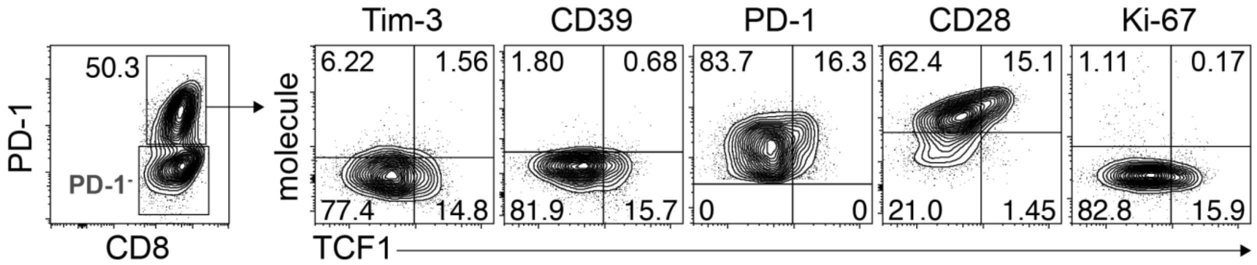

Supplemental Figure 6. Phenotypic characteristics of CD8 TILs in RCC. Phenotype of PD-1+ CD8 T cells among RCC TILs from individuals with no Tim-3^{hi} cells. Data are representative of 2 individuals.

Supplementary Figure 7. Lee JH, Lee KM, and et al.

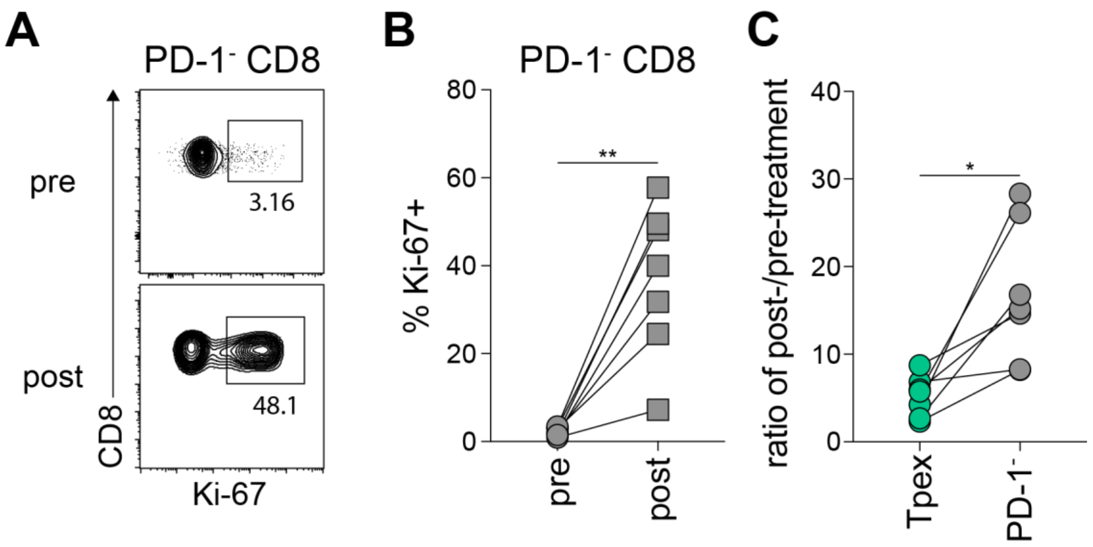

Supplemental Figure 7. Tpex cells in human RCC tumors have compromised proliferative capacity in response to *ex vivo* treatment of IL-15 compared with PD-1- CD8 TILs. The same experimental setup as described in Figure 7H-J was used. (**A, B**) Representative flow plots (**A**) and a summary graph (**B**) showing the frequency of Ki-67+ cells among PD-1- CD8 T cells from RCC tumors. (**C**) Comparison of IL-15-driven proliferation between Tpex cells (which is shown in Figure 7H-J) and PD-1- CD8 T cells from the same patients before and after 3 days of *ex vivo* culture with IL-15. Data were combined from two independent experiments with samples from 7 patients. *$P < 0.05$; **$P < 0.01$ (Student's *t*-test, paired).
